# Weigh-In to Fight Night: Dietary Strategies of Professional MMA Fighters

**DOI:** 10.3390/nu18172880

**Published:** 2026-09-02

**Authors:** Cassandra Evans, Minh K. Chau, Louis Tonnel, Jack Landmesser, Charles Stull, Duncan N. French, Jose Antonio, Corey A. Peacock

**Affiliations:** 1 Dr. Kiran C. Patel College of Osteopathic Medicine, Fight Science Laboratory, Nova Southeastern University, Davie, FL 33328, USA; jl3533@mynsu.nova.edu (J.L.); jose.antonio@nova.edu (J.A.); cpeacock@nova.edu (C.A.P.); 2 Department of Psychology and Neuroscience, Nova Southeastern University, Davie, FL 33328, USA; mc4076@nova.edu; 3 Dr. Kiran C. Patel College of Allopathic Medicine, Nova Southeastern University, Davie, FL 33328, USA; lt1228@mynsu.nova.edu; 4 Ultimate Fighting Championship Performance Institute (UFC PI), Las Vegas, NV 89118, USA; charles.stull@email.ufc.com (C.S.); duncan.french@ufc.com (D.N.F.); 5 School of Behavioural and Health Sciences, Australian Catholic University, Melbourne, VIC 3065, Australia

**Keywords:** mixed martial arts, weight cutting, rapid weight loss, refeeding, rehydration

## Abstract

**Background/Objectives:** Professional mixed martial arts (MMA) athletes routinely partake in practices to elicit rapid weight loss before official weigh-in, followed by aggressive rehydration and nutritional recovery before competition. Although weight-cutting strategies have been widely described, there is limited information characterizing nutrient intake, food group selection, and fluid consumption during the pre-weigh-in, post-weigh-in, and fight-day periods. This cross-sectional study examined self-reported dietary intake of professional MMA fighters during the 24 h before weigh-in, post-weigh-in, and on fight day. **Methods:** Participants self-reported all foods and beverages consumed during three distinct competition phases: pre-weigh-in, post-weigh-in, and fight day. Changes in energy, macronutrient, fiber, sodium, and fluid intake across competition phases were evaluated using linear mixed-effects models. **Results:** The pre-weigh-in period was characterized by severe carbohydrate and fiber restriction with reduced fluid intake, whereas post-weigh-in intake shifted toward aggressive carbohydrate, fluid, and sodium replenishment alongside reduced fat consumption. Food group analysis demonstrated systematic elimination of vegetables, dairy products, legumes, whole grains, fried foods, and other high-fiber foods before weigh-in, with continued restriction of high-fat and high-fiber foods throughout the recovery period. **Conclusions:** These findings provide a comprehensive characterization of nutrient intake, food selection, and fluid consumption across the final stages of professional MMA competition preparation.

## 1. Introduction

Since the early 1990s, Mixed Martial Arts (MMA) has evolved into a globally regulated sport that integrates multiple disciplines, including wrestling, Brazilian Jiu-Jitsu, Muay Thai, and kickboxing [1]. Its rapid rise in popularity led to the establishment of several professional organizations, with the Ultimate Fighting Championship (UFC) having established itself as the dominant organization in mixed martial arts, exercising substantial market control over elite fighters [2]. Fighters compete within established weight classes, and bouts typically consist of three five-minute rounds for non-championship fights and five rounds for championship contests.

A defining characteristic of MMA and other combat sports is the cyclical process of rapid weight loss (RWL) prior to official weigh-in, followed by rapid weight gain (RWG) in the 24–36 h preceding competition. Compared to other combat sports, MMA athletes exhibit some of the largest magnitudes of RWG, averaging 7.4 ± 2.8 kg [3]. More than 25% of male fighters and 33% of female fighters regain over 10% of their body mass between weigh-in and competition [4], with some athletes exceeding their pre-cut weight [5]. Consequently, fighters often enter competition at a body mass equivalent to one or two weight classes above their official division [6].

The motivations underlying RWG are multifactorial. Although rehydration and replenishment of depleted substrates are necessary after rapid weight loss, greater post-weigh-in mass is widely perceived to confer advantages in strength, leverage, and power [7]. However, evidence regarding the competitive benefit of RWG remains inconsistent. Some studies report that winners demonstrate significantly greater RWG than losers [8], whereas more recent analyses have found no significant association between regaining ≥10% of body mass and fight outcome [9], nor between body mass differences and method of victory [6]. Beyond physiological considerations, the weight-cutting and regain cycle is deeply embedded in combat sports culture, often symbolizing preparedness, discipline, and competitive confidence [7].

Despite the well-documented prevalence and magnitude of RWL and RWG, few studies have characterized what combat athletes actually consume during these phases. Existing literature has identified common strategies for manipulating body mass, including carbohydrate restriction, fluid manipulation, sodium restriction, and other acute weight-cutting practices. However, comparatively little is known about the specific food selection patterns athletes use to implement these strategies during the final stages of competition preparation. Characterizing the foods and beverages consumed before weigh-in and during post-weigh-in recovery may provide more practical insight into how athletes translate these nutritional strategies into real-world practice. Therefore, the purpose of this study was to characterize energy and nutrient intake, food group selection, and fluid consumption across the pre-weigh-in, post-weigh-in recovery, and fight-day phases in professional MMA athletes and to examine whether these practices differed by weight class. To our knowledge, no previous study has comprehensively characterized both macronutrient intake and food group selection across all three competition phases in professional MMA athletes.

## 2. Materials and Methods

This study used a cross-sectional survey design to examine the dietary intake of combat sport athletes during rapid weight loss (RWL) and post-weigh-in/fight-day periods. Participants completed a single survey in which they reported dietary intake across the pre-weigh-in, post-weigh-in, and fight-day phases. All participants were adults competing in UFC across different weight classes. The study was reviewed by the Institutional Review Board at Nova Southeastern University (Protocol 2026-146) and determined to be exempt. The requirement for informed consent was waived under the approved exempt protocol. Participants were recruited through online distribution of a survey link. Eligibility criteria included: (1) current participation in a combat sport requiring weight-class competition and (2) experience with at least one competitive weigh-in. Individuals were excluded if they were not actively competing or did not complete all required dietary recall components. Eligibility was assessed via self-report.

### 2.1. Dietary Intake

Dietary intake data were collected using an online survey platform (Qualtrics, Provo, UT, USA) across three distinct competition phases: (1) pre-weigh-in, representing intake during the 24 h immediately preceding official weigh-in; (2) post-weigh-in, representing intake following official weigh-in through the remainder of weigh-in day; and (3) fight day, representing intake specifically reported for the day of competition. When dietary records included timing information relative to weigh-in (e.g., at weigh-in, <2 h post-weigh-in, or later on weigh-in day), these entries were categorized within the post-weigh-in period, whereas foods and beverages specifically identified as fight-day intake were categorized separately.

Food records were subsequently coded and entered into the Nutritionix database (Nutritionix, New York, NY, USA) by three members of the research team to calculate total energy intake (kcal), macronutrient intake (g), dietary fiber (g), fluid intake (fl oz), and sodium (mg). When multiple database entries were available for a reported food, the entry most closely corresponding to the food description, brand, and portion information provided in the original record was selected. Nutritionix was selected because of its use within UFC organizations and to align the nutrient analysis with real-world athlete practice.

### 2.2. Statistical Analysis

Food intake data were categorized into seven predefined food groups based on primary macronutrient composition: protein, fat, simple carbohydrate, fruit, vegetables, complex carbohydrate, and supplement. Categories were assigned using prespecified keyword-matching rules implemented in R with the stringr package. Keywords included representative food items within each category (e.g., eggs, chicken, and beef for protein; rice, pasta, and potatoes for complex carbohydrate; and Vitargo and Oral Rehydration Solution (ORS) for supplements). Fruit was classified separately from simple carbohydrates because of its consistent reporting across time points. Frequency counts were then used to quantify the number of reported food items within each category. Counts represent food-item occurrences rather than the number of participants, and individual participants could contribute multiple items to a given category.

To examine within-person changes across time points, separate linear mixed-effects models (LMMs) were fitted for each dietary outcome, with time point included as a fixed effect and participant as a random intercept. Omnibus effects were evaluated using Type III *F*-tests, and effect sizes were quantified using partial eta-squared ($\eta_{p}^{2}$). Significant omnibus effects were followed by Tukey’s honestly significant difference (HSD) post hoc pairwise comparisons. Repeated measures Cohen’s *d*_rm_ and bootstrap 95% confidence intervals (1000 iterations) were calculated to quantify pairwise effect sizes. Participants with missing data at one time point were retained in analyses of their available data at other time points, and missing values were not imputed.

To examine whether weight class predicted pre-weigh-in nutritional intake, separate one-way analyses of variance (ANOVAs) were conducted for each dietary outcome. Significant omnibus effects were followed by Tukey-adjusted post hoc comparisons, with Cohen’s *d* reported as the effect size for pairwise comparisons.

To control the familywise error rate at *α* = 0.05 across the omnibus analyses, a Bonferroni-adjusted significance threshold of *α* = 0.0036 was applied across the 14 omnibus tests (seven LMMs and seven one-way ANOVAs). Post hoc pairwise comparisons retained a significance level of *α* = 0.05 following Tukey adjustment.

## 3. Results

### 3.1. Participants

A total of *N* = 107 professional mixed martial arts (MMA) fighters (*n*_female_ = 15, *n*_male_ = 92) across eight UFC weight classes were included in the analysis. The mean age of the full sample was 31.1 years (*SD* = 4.35). Male fighters had a mean age of 31.2 years (*SD* = 4.37), whereas female fighters had a mean age of 35 years (*SD* = 4.29). Participant characteristics are presented in Table 1.

### 3.2. Food Groups

During the pre-weigh-in period, protein was most frequently reported (*n* = 328), followed by fat (*n* = 241), fruit (*n* = 107), and simple carbohydrate (*n* = 107) (Table 2). Complex carbohydrates were minimally reported (*n* = 6), while supplements and vegetables were not reported (*n* = 0). Post-weigh-in, complex carbohydrate was the most frequently reported category (*n* = 279), followed by protein (*n* = 198), simple carbohydrate (*n* = 172), fruit (*n* = 107), and supplements (*n* = 107). Fat decreased from 241 pre-weigh-in reports to 0 post-weigh-in reports. Fruit remained stable (*n* = 107 at both time points), while vegetables were not reported at either time point.

### 3.3. Changes in Nutritional Intake Across Competition Phases

Dietary intake data were available for 107 participants at pre-weigh-in, 106 participants at post-weigh-in, and 87 participants on fight day. Linear mixed-effects models (LMMs) incorporated all available observations for each outcome. LMMs revealed significant main effects of time point for all seven dietary outcomes (all Bonferroni-corrected *p*s < 0.0036; Table 3). Effect sizes were uniformly large, ranging from $\eta_{p}^{2}$ = 0.26 for protein to $\eta_{p}^{2}$ = 0.93 for carbohydrate. Descriptive statistics by competition phase are presented in Table 3.

Energy intake differed across all three competition phases, with the highest intake occurring post-weigh-in (2501 ± 243 kcal), followed by fight day (1985 ± 418 kcal), and pre-weigh-in (1751 ± 573 kcal; *d*_rm_ = 0.23–1.41). Carbohydrate intake increased from 55.1 ± 15.2 g pre-weigh-in to 345.9 ± 57.7 g post-weigh-in before declining to 278 ± 70.9 g on fight day, with all three phases differing significantly (*d*_rm_ = 0.88–3.56). In contrast, fat intake was highest pre-weigh-in (137 ± 46 g) and significantly lower on both fight day (49.4 ± 15.9 g) and post-weigh-in (56.5 ± 11.8 g), which did not differ from one another (*d*_rm_ = 1.27–1.45).

Protein intake was significantly lower pre-weigh-in (75.9 ± 35.2 g) than on fight day (99.2 ± 19.8 g) and post-weigh-in (104 ± 24.6 g), whereas the latter two phases did not differ (*d*_rm_ = 0.36–0.44). Dietary fiber intake increased from 4.53 ± 2.02 g pre-weigh-in to 17.0 ± 3.71 g post-weigh-in and decreased to 13.9 ± 4.36 g on fight day, with all three phases differing significantly (*d*_rm_ = 0.72–2.14). Water intake increased approximately sixfold from pre-weigh-in to post-weigh-in (38.6 ± 31.7 vs. 223 ± 54.2 fl oz; *d*_rm_ = 1.73), whereas sodium intake increased approximately sevenfold (781 ± 404 vs. 5463 ± 1654 mg; *d*_rm_ = 2.12).

### 3.4. Weight-Class Differences at Pre-Weigh-In

One-way ANOVAs indicated significant weight-class differences for energy, protein, fat, and water (all *p*s < 0.0036; η^2^ = 0.29–0.43; Table 4). Carbohydrate, fiber, and sodium did not reach the Bonferroni-corrected significance threshold. Descriptive statistics by weight class and associated post hoc comparisons are presented in Table 4 and Table 5.

Tukey HSD post hoc comparisons indicated that Light Heavyweight fighters consumed significantly more energy, protein, fat, and water than all other weight classes (*d*s = 1.51–2.77). Middleweight fighters consumed significantly more energy than Bantamweight, Flyweight, Strawweight, and Welterweight fighters; significantly more protein than Bantamweight, Flyweight, and Welterweight fighters; and significantly more fat than Bantamweight, Flyweight, Strawweight, and Welterweight fighters (*d*s = 0.98–1.66). No other significant pairwise differences were observed. Carbohydrate, dietary fiber, and sodium intake did not differ significantly among weight classes (Table 4).

## 4. Discussion

The present study provides a comprehensive characterization of dietary intake across three phases of MMA competition in 107 professional fighters. The results reveal a highly structured, phase-dependent nutritional pattern consistent with deliberate weight management and tactical refeeding strategies.

### 4.1. Food Groups

Analysis of dietary logs demonstrated that food selection during the final stages of competition preparation varied across competition phases, with athletes eliminating or restricting specific food groups. These variations appear to align with the physiological objectives of each phase. During the pre-weigh-in period, protein and fat were the most frequently reported food categories, while fruit and simple carbohydrates were also consistently reported (Table 2). Conversely, complex carbohydrates were minimally reported and vegetables and supplements were absent from dietary records. These findings are consistent with previous reports demonstrating that combat sport athletes modify food selection during competition preparation, often reducing carbohydrate- and fiber-rich foods while maintaining relatively high protein intake [10]. The near absence of complex carbohydrates and complete absence of vegetables during the pre-weigh-in period further characterize the restrictive dietary patterns frequently used to elicit RWL. The present study extends these observations by reporting that several of these food groups were not simply reduced but were completely eliminated during the final 24 h pre-weigh-in period. The complete elimination of several food groups represents one of the more novel findings of the present study. Previous studies have generally reported reductions in these foods, whereas the present data demonstrate that professional MMA athletes often remove them entirely during the final 24 h before weigh-in. This finding provides practitioners with a clearer picture of what athletes are actually doing during the most aggressive phase of weight cutting.

The specific food selection reflects multiple physiological objectives beyond energy restriction alone. Consistent with current combat sport nutrition recommendations, rapid weight loss is achieved through coordinated manipulation of dietary composition, gastrointestinal contents, glycogen stores, and body water rather than caloric restriction alone [11,12]. The minimal reporting of complex carbohydrates is consistent with the substantial carbohydrate restriction observed in the quantitative nutrient analysis, while the absence of vegetables may contribute to the very low dietary fiber intake observed during this period. Reducing dietary fiber and other sources of gastrointestinal residue can decrease intestinal contents and non-functional body mass before official weigh-in. These dietary modifications complement carbohydrate restriction by minimizing gastrointestinal contents while facilitating rapid weight loss. These dietary patterns are consistent with established RWL practices involving manipulation of carbohydrate intake and gastrointestinal contents.

Following weigh-in, food selection shifted toward carbohydrate-rich foods and supplements, likely related to optimizing recovery after extreme restriction. Complex carbohydrate became the most frequently reported food category, simple carbohydrate intake increased, and supplements were frequently reported, while protein and fruit continued to be commonly reported. In contrast, fat was no longer reported and vegetables remained absent. This pattern is consistent with a transition away from weight reduction toward dietary practices that may facilitate gastric emptying, minimize gastrointestinal distress, and promote rapid nutrient and fluid delivery during the limited recovery window before competition [11]. These findings suggest practitioners should prioritize rapidly digestible carbohydrate sources while delaying reintroduction of higher-fat and higher-fiber foods. The gradual relaxation of dietary restrictions as fight day approached further suggests that athletes progressively transitioned from aggressive recovery strategies toward a more typical competition-day diet while continuing to prioritize gastrointestinal comfort.

### 4.2. Changes in Nutritional Intake Across Competition Phases

The dietary patterns reported during the different time points are consistent with strategies that professional MMA athletes use to elicit RWL through manipulation of glycogen stores, gastrointestinal contents, and body water rather than relying solely on energy restriction [10,12,13].

The carbohydrate intake patterns observed during the pre-weigh-in period closely align with contemporary rapid weight-cutting strategies described in the combat sport literature (Table 3). Although current sports nutrition guidelines recommend carbohydrate intakes of 6–12 g/kg/day depending on training volume [14], MMA athletes in the present study implemented much more aggressive carbohydrate restriction immediately before weigh-in. This strategy is used to deplete glycogen stores and the associated water required for glycogen storage. Dietary fiber intake was similarly reduced, consistent with minimizing gastrointestinal residue and intestinal mass before weigh-in [11]. The ISSN Position Stand recommends maintaining carbohydrate intake above 3–4 g/kg/day during prolonged weight-cutting or chronic weight loss phases where athletes typically undergo reduction in fat mass whilst preserving fat free mass. This is in contrast to the acute glycogen depletion strategies utilized for acute weight-cutting whereby fewer than 50 g/day may be consumed in the days leading up to weigh-ins [11]. The average carbohydrate intake observed in the present study closely aligns with these acute restriction strategies, while the near-zero variance in carbohydrate and fiber intake across weight classes suggests that severe carbohydrate restriction has become a near-universal practice among professional MMA athletes. During this period, reduced carbohydrate intake was accompanied by relatively high fat intake, suggesting that athletes compensated for reduced carbohydrate availability by increasing dietary fat to maintain energy intake while preserving glycogen depletion. Based on our results, the carbohydrate intake pattern demonstrates that along with water manipulation strategies, carbohydrate manipulation is also a primary nutritional strategy taking place during both the weight-loss and recovery phase.

Reported sodium intakes during the pre-weigh-in period further support the concept that professional MMA athletes use multiple nutritional strategies to manipulate body mass before official weigh-in (Table 3). Although the rationale for sodium restriction was not assessed, these findings align with the current literature, which describes reducing intake as another component of RWL. Reducing sodium intake is thought to limit extracellular water retention by minimizing sodium-driven fluid retention and the associated increase in body mass. The sodium intake patterns observed in the present study suggest that professional MMA athletes may routinely implement more aggressive sodium restriction than is currently recommended. The aforementioned study findings demonstrate the multiple strategies used to achieve RWL.

Post-weigh-in intake demonstrates that athletes rapidly transition from weight-loss strategies to recovery-focused nutrition aimed at restoring glycogen, fluid balance, and competition readiness. Following weigh-in, athletes demonstrated substantial increases in carbohydrate, fluid, and sodium intake consistent with the deliberate strategies used to restore glycogen stores, promote rehydration, and replenish electrolytes before competition [11,12]. Current recommendations suggest carbohydrate intakes of 4–7 g/kg following moderate glycogen depletion and 8–12 g/kg following substantial glycogen depletion to maximize glycogen restoration [15]. However, the relatively short 24–36 h recovery window between weigh-in and competition presents a considerable challenge to achieving complete glycogen repletion. Martínez-Aranda et al. [13] reported that combat sport athletes rapidly reintroduce carbohydrate-rich foods and fluids following weigh-in to facilitate recovery, although the available recovery period may still be insufficient to fully restore glycogen stores and hydration status. Rapid carbohydrate reintroduction may also increase the risk of gastrointestinal distress, emphasizing the importance of both the quantity and type of carbohydrate consumed [16]. Consequently, practical recommendations include prioritizing high-glycemic-index carbohydrate sources, utilizing oral rehydration solutions [17], and consuming multiple carbohydrate sources such as glucose and fructose to maximize intestinal carbohydrate absorption through separate transport pathways [18]. Although athletes demonstrated aggressive refeeding, the observed intakes suggest that complete restoration of glycogen stores may still be difficult within the available recovery window.

Fluid intake also increased substantially following weigh-in, reflecting the transition from intentional dehydration to aggressive rehydration. Current guidelines recommend replacing 125–150% of fluid losses incurred during weight cutting [19], with sodium concentrations adjusted according to the severity of dehydration [11,19]. Given that MMA athletes may lose approximately 4.4% of body mass through dehydration during the 24 h preceding weigh-in [8], the fluid intake observed in the present study appears largely consistent with recommended rehydration strategies.

The post-weigh-in period was also characterized by reduced fat intake despite marked increases in total energy and carbohydrate consumption. Lower fat intake during this phase may reduce gastrointestinal discomfort before competition, an important consideration given that competition-related anxiety may further impair gastric emptying and exacerbate gastrointestinal symptoms [20,21]. The magnitude of these shifts, all characterized by large effect sizes, underscores the extent to which dietary manipulation is practiced in professional MMA competition preparation.

Interestingly, total energy intake, carbohydrate, and fiber intake on fight day decreased slightly, likely reflecting both continued refueling and practical constraints around competition timing. Although carbohydrate intake was still substantially higher than during the pre-weigh-in period, fight-day intake remained below current recommendations for optimal glycogen restoration [15]. Based on current carbohydrate recommendations for glycogen restoration, this finding suggests that, despite aggressive post-weigh-in refueling, professional MMA fighters may not fully restore glycogen stores before competition. Consequently, optimizing carbohydrate intake during the limited recovery period between weigh-in and competition may represent an opportunity for sports dietitians and performance practitioners to further enhance competition readiness. This finding suggests that fight-day nutrition may represent one of the greatest opportunities for practitioner intervention. While athletes appear highly proficient at weight loss and immediate post-weigh-in refeeding, nutritional intake during competition day itself may remain suboptimal.

### 4.3. Weight-Class Differences at Pre-Weigh-In

Despite substantial differences in body size across weight classes, professional MMA athletes appeared to adopt remarkably similar acute weight-cutting strategies. Rather than adjusting intake (carbohydrate, dietary fiber, and sodium restriction) according to body mass, athletes across divisions converged on nearly identical dietary patterns during the final stages of rapid weight loss. This consistency extended beyond nutrient intake to food selection, with fighters across all weight classes consuming virtually the same types of foods while eliminating similar food groups. These findings suggest that the primary objective is not meeting individualized nutritional requirements but achieving a common goal of rapid weight loss using similar practices, including glycogen depletion and reductions in gastrointestinal contents and body water to achieve the desired weight.

As expected, heavier weight classes consumed more total energy, protein, fat, and water during the pre-weigh-in period, reflecting greater body size and absolute energy requirements. In contrast, carbohydrate intake remained remarkably consistent across weight classes despite substantial differences in body mass. This finding suggests that professional MMA athletes may prioritize achieving a similar degree of glycogen depletion during acute weight cutting rather than adjusting carbohydrate intake according to body size. Previous work has demonstrated that rapid weight-cutting practices are widely employed across professional MMA weight classes, with athletes across nearly all divisions engaging in substantial weight loss before official weigh-in followed by rapid weight regain prior to competition [8]. The present findings extend these observations by demonstrating that the nutritional strategies used to achieve these weight changes are similarly consistent across weight classes, particularly with respect to carbohydrate restriction. This interpretation is further supported by evidence that acute weight-cutting strategies rely primarily on manipulation of glycogen, body water, and intestinal contents, with low-carbohydrate diets representing a central component of rapid weight loss [3].

### 4.4. Limitations

Several limitations warrant consideration. The observational design precludes causal inference regarding the effects of these nutritional practices on performance outcomes. The sample was predominantly male, limiting generalizability to female competitors. Dietary data relied on retrospective self-report, which introduces potential recall and social desirability biases. Reported foods, fluids, and portion sizes could not be independently verified, including whether all reported items were consumed in their entirety. Additionally, nutrient intake was estimated using a commercial nutrient database and may be subject to variability related to the specificity of participant-reported foods and portion sizes and the corresponding database entries selected for analysis. Fighters’ competition weight class was collected rather than individual body weight at each time point; therefore, intake relative to body mass could not be calculated. Differences in absolute intake between weight classes may be influenced by differences in body size. Finally, actual weight loss and regain, hydration status, and glycogen stores were not directly measured; therefore, physiological interpretations related to these outcomes are based on the observed dietary practices and existing literature. Future research should examine the longitudinal health implications of repeated acute weight cycling and evaluate the efficacy of refueling protocols in relation to competition performance.

## 5. Conclusions

In summary, professional MMA fighters use highly structured nutritional strategies to support rapid weight loss and subsequent recovery before competition. Weight cutting involved coordinated manipulation of carbohydrate, fat, fiber, and fluid intake rather than energy restriction alone, while post-weigh-in nutrition focused on restoring carbohydrate, fluid, and electrolyte stores. Although heavier fighters consumed greater absolute amounts of energy and macronutrients, the overall dietary strategy was remarkably consistent across weight classes. Collectively, these findings provide one of the first detailed descriptions of how professional MMA athletes manipulate both nutrient intake and food selection across the final stages of competition preparation. The results offer practitioners evidence-based insight into current athlete behaviors while identifying several opportunities to optimize post-weigh-in recovery and fight-day nutrition.

## Figures and Tables

**Table 1:** Sample Characteristics by UFC Weight Class.

Weight Class	*N*	Male	Female	Mean ± SD Age (Years)
Strawweight	5	—	5	31.2 ± 6.06
Flyweight	12	6	6	28.7 ± 3.20
Bantamweight	19	15	4	30.8 ± 3.95
Featherweight	10	10	—	30.9 ± 5.53
Lightweight	6	6	—	31.0 ± 2.45
Welterweight	18	18	—	32.7 ± 5.48
Middleweight	25	25	—	31.1 ± 3.99
Light Heavyweight	12	12	—	31.9 ± 3.53
Total	107	92	15	31.1 ± 4.35

Age is reported as mean ± SD. Weight class represents the UFC weight class in which participants reported competing. “—” indicates no competitors or missing age data for that sex within the respective weight class.

**Table 2:** Food Groups.

Food Category	Pre-Weigh-In (*n*)	Post-Weigh-In (*n*)
Protein	328	198
Fat	241	0
Simple Carbohydrate	107	172
Fruit	107	107
Vegetables	0	0
Complex Carbohydrate	6	279
Supplement	0	107

**Table 3:** Dietary Intake Before Weigh-In, After Weigh-In, and on Fight Day.

Outcome	Pre-Weigh-In Mean ± SD (*n* = 107)	Post-Weigh-In Mean ± SD (*n* = 106)	Fight Day Mean ± SD (*n* = 87)	*F* (*df*_1_*, df*_2_)	$\eta_{p}^{2}$
Energy (kcal)	1751 ± 573.0 ^c^	2501 ± 243 ^a^	1985 ± 418 ^b^	*F*(2, 197) = 85.58 *	0.46
Carbohydrate (g)	55.1 ± 15.2 ^c^	345.9 ± 57.7 ^a^	278 ± 70.9 ^b^	*F*(2, 201) = 1331 *	0.93
Protein (g)	75.9 ± 35.2 ^b^	104 ± 24.6 ^a^	99.2 ± 19.8 ^a^	*F*(2, 192) = 32.92 *	0.26
Fat (g)	137 ± 46 ^a^	56.5 ± 11.8 ^b^	49.4 ± 15.9 ^b^	*F*(2, 197) = 309.78 *	0.76
Dietary Fiber (g)	4.53 ± 2.02 ^c^	17.0 ± 3.71 ^a^	13.9 ± 4.36 ^b^	*F*(2, 202) = 509.37 *	0.83
Water (fl oz)	38.6 ± 31.7 ^b^	223 ± 54.2 ^a^	—	*F*(1, 211) = 876.25 *	0.81
Sodium (mg)	781 ± 404 ^b^	5463 ± 1654 ^a^	—	*F*(1, 107) = 857.77 *	0.89

Data are presented as mean ± SD for descriptive purposes. F-statistics were obtained from linear mixed-effects models. Within each row, superscripts denote Tukey HSD homogeneous subsets (α = 0.05): values sharing a letter do not differ significantly. Water and sodium were assessed at pre-weigh-in and post-weigh-in only. * *p* < Bonferroni-corrected α = 0.0036. *n* represents the number of unique participants with available data for the respective outcome at each competition phase. Linear mixed-effects models used all available observations and retained participants with incomplete repeated measurements.

**Table 4:** Pre-Weigh-In Nutritional Intake by UFC Weight Class: Means ± SD with Inferential Statistics.

Outcome	STW (*n* = 5)	FW (*n* = 12)	BW (*n* = 19)	FEW (*n* = 10)	LW (*n* = 6)	WW (*n* = 18)	MW (*n* = 25)	LHW (*n* = 12)	*F*(*df*_1_ , *df*_2_)	*η* ^2^	95% CI
Energy (kcal)	1319 ± 291	1364 ± 296	1491 ± 415	1747 ± 530	1599 ± 593	1536 ± 198	2046 ± 587 ^b^	2522 ± 508 ^a^	*F*(7, 99) = 9.87 *	0.41	[0.26, 1.0]
Carbohydrate (g)	52.2 ± 0.45	52.1 ± 0.29	53.4 ± 4.30	52.4 ± 0.52	52.3 ± 0.52	52.6 ± 0.62	57.4 ± 16.5	64.8 ± 38.1	*F*(7, 99) = 1.06	0.07	[0.0, 1.0]
Protein (g)	52.8 ± 22.4	55.9 ± 23.4	57.7 ± 22.8	71.8 ± 28.2	71.5 ± 40.1	63.3 ± 15.0	90.3 ± 33.9 ^c^	129 ± 31.7 ^a^	*F*(7, 99) = 10.54 *	0.43	[0.28, 1.0]
Fat (g)	101 ± 21.2	105 ± 24.1	118 ± 35.9	139 ± 46.4	123 ± 47.3	119 ± 15.9	162 ± 46.3 ^b^	198 ± 36.0 ^a^	*F*(7, 99) = 9.96 *	0.41	[0.27, 1.0]
Dietary Fiber (g)	4.30 ± 0.0	4.30 ± 0.0	4.30 ± 0.0	4.30 ± 0.0	4.30 ± 0.0	4.30 ± 0.0	4.32 ± 0.23	6.33 ± 5.92	*F*(7, 99) = 1.60	0.10	[0.0, 1.0]
Water (fl oz)	27.0 ± 22.7	23.7 ± 8.40	33.4 ± 25.6	30.4 ± 16.0	22.5 ± 8.73	33.3 ± 15.7	42.6 ± 35.2	84.6 ± 49.1 ^a^	*F*(7, 98) = 5.62 *	0.29	[0.13, 1.0]
Sodium (mg)	643 ± 97	641 ± 81	671 ± 104	721 ± 94	738 ± 186	678 ± 91	982 ± 763	1023 ± 233	*F*(7, 99) = 2.32	0.14	[0.01, 1.0]

Values are mean ± SD. One-way ANOVAs compared dietary intake among UFC weight classes during the pre-weigh-in period. Superscripts denote significant Tukey HSD pairwise comparisons (α = 0.05): ^a^ = significantly greater than all other weight classes (Light Heavyweight); ^b^ = significantly greater than Bantamweight, Flyweight, Strawweight, and Welterweight (Middleweight for energy and fat); ^c^ = significantly greater than Bantamweight, Flyweight, and Welterweight (Middleweight for protein). Carbohydrate, dietary fiber, and sodium did not reach the Bonferroni-corrected significance threshold (*α* = 0.0036); therefore, no superscripts are shown. STW = Strawweight; FW = Flyweight; BW = Bantamweight; FEW = Featherweight; LW = Lightweight; WW = Welterweight; MW = Middleweight; LHW = Light Heavyweight. * = *p* < Bonferroni-corrected α = 0.0036.

**Table 5:** Post Hoc Tukey HSD Pairwise Comparisons for UFC Weight Class (One-Way ANOVAs).

Outcome	Comparison	Mean_Diff_	*t*	*d* [95% CI]
Energy (kcal)	LHW vs. Bantamweight	1031	4.55	2.27 [1.47, 3.06]
	LHW vs. Featherweight	775	3.11	1.71 [0.82, 2.59]
	LHW vs. Flyweight	1158	4.78	2.55 [1.66, 3.43]
	LHW vs. Lightweight	923	3.30	2.03 [1.0, 3.06]
	LHW vs. Strawweight	1203	4.23	2.65 [1.53, 3.77]
	LHW vs. Welterweight	986	4.36	2.17 [1.37, 2.97]
	MW vs. Bantamweight	555	3.17	1.22 [0.59, 1.85]
	MW vs. Flyweight	682	3.51	1.50 [0.77, 2.23]
	MW vs. Strawweight	727	3.03	1.60 [0.60, 2.60]
	MW vs. Welterweight	510	2.93	1.12 [0.49, 1.75]
Protein (g)	LHW vs. Bantamweight	71.4	5.23	2.59 [1.77, 3.41]
	LHW vs. Featherweight	57.3	3.80	2.08 [1.18, 2.98]
	LHW vs. Flyweight	73.2	5.25	2.66 [1.76, 3.55]
	LHW vs. Lightweight	57.6	3.42	2.09 [1.06, 3.13]
	LHW vs. Middleweight	38.8	3.50	1.41 [0.68, 2.13]
	LHW vs. Strawweight	76.3	4.45	2.77 [1.64, 3.90]
	LHW vs. Welterweight	65.8	5.02	2.39 [1.58, 3.20]
	MW vs. Bantamweight	32.6	3.53	1.18 [0.56, 1.81]
	MW vs. Flyweight	34.4	3.40	1.25 [0.53, 1.97]
	MW vs. Welterweight	27.0	2.97	0.98 [0.35, 1.61]
Fat (g)	LHW vs. Bantamweight	80.0	4.47	2.21 [1.42, 3.01]
	LHW vs. Featherweight	59.0	2.89	1.62 [0.74, 2.50]
	LHW vs. Flyweight	93.0	5.01	2.55 [1.66, 3.43]
	LHW vs. Lightweight	75.0	3.43	2.06 [1.03, 3.10]
	LHW vs. Strawweight	97.0	3.74	2.65 [1.53, 3.78]
	LHW vs. Welterweight	79.0	4.43	2.16 [1.36, 2.96]
	MW vs. Bantamweight	44.0	3.04	1.22 [0.59, 1.84]
	MW vs. Flyweight	57.0	3.68	1.55 [0.82, 2.28]
	MW vs. Strawweight	61.0	2.89	1.66 [0.66, 2.65]
	MW vs. Welterweight	43.0	3.09	1.16 [0.53, 1.80]
Water (fl oz)	LHW vs. Bantamweight	51.2	3.41	1.85 [1.05, 2.64]
	LHW vs. Featherweight	54.2	3.34	1.95 [1.04, 2.86]
	LHW vs. Flyweight	60.9	3.97	2.20 [1.31, 3.08]
	LHW vs. Lightweight	62.1	3.49	2.24 [1.18, 3.29]
	LHW vs. Middleweight	42.0	3.05	1.51 [0.77, 2.26]
	LHW vs. Strawweight	57.6	2.95	2.08 [0.97, 3.19]
	LHW vs. Welterweight	51.3	3.41	1.85 [1.05, 2.65]

Only significant pairwise contrasts (Tukey HSD, *α* = 0.05) are reported for outcomes with significant omnibus effects (kcal, protein, fat, water). Cohen’s *d* and bootstrap 95% confidence intervals (CIs) are provided. Non-significant outcomes (carbohydrate, fiber, and sodium) are omitted. LHW = Light Heavyweight; MW = Middleweight.

## Data Availability

The raw data supporting the conclusions of this article will be made available by the authors on request.
